# Barriers to Surgical Innovation Research: A Canadian Study on Public Funding Trends

**DOI:** 10.1177/15533506221085469

**Published:** 2022-04-15

**Authors:** Rachel Wang, Kaija P Kaarid, May Sanaee

**Affiliations:** 1 Department of Obstetrics and Gynecology, 3158 University of Alberta, Edmonton, Canada; 2 Department of Obstetrics and Gynecology, 7512 Memorial University of Newfoundland, St. John’s, Canada

**Keywords:** surgical innovation, surgical invention, surgical invention

## Abstract

**Background:**

A decline in research funding has been cited as a potential cause for limited surgical innovation in the United States. We aim to understand if this is a North American phenomenon and explore whether a lack of public funding is a barrier to surgical innovation in Canada.

**Methods:**

Publicly available funding data from Canadian Institutes of Health Research (CIHR) were reviewed from 2008 to 2019 to determine the yearly funding distributed to surgical departments. Surgical innovation studies were identified and total yearly funding was calculated. All amounts were adjusted for inflation to reflect 2019 Canadian dollar value.

**Results:**

From 2008 to 2019, surgical departments were granted 1.82–4.70% of total CIHR funding. In total, 902 grants were allocated to surgical departments and 126 (14.0%) met criteria for surgical innovation. Surgical innovation research was allocated a total annual amount ranging from 1.52 to 9.01 million CAD. There appears to be an upward trend in public funding for surgical innovation over this time period.

**Discussion:**

Contrary to the landscape in the United States, there is no evidence of decreasing trends in public funding for surgical innovation in Canada. Considerations should be given to other potential barriers precluding surgeons from participating in innovation.

**Conclusion:**

Only a small percentage of research dollars to departments in Canada are spent on innovation research, despite an overall increasing trend in total public research funding over the past 10 years. We need to foster an environment in which surgical innovation is encouraged through medical curriculum changes, multidisciplinary collaboration opportunities, and dedicated faculty resources.

## Background

Modern laparoscopy, with a forward-viewing scope and improved lens, was first developed in 1929. Since then, improvements in minimally invasive surgery have paralleled other technological advances, and many surgical subspecialties have adopted this innovative approach. An innovative procedure in surgery is not simply an improvement, and it is described as “a new or modified surgical procedure that differs from currently accepted local practice, the outcomes of which have not been described, and which may entail risk to the patient.”^1^ Surgical innovation is a broad term, which can include new technologies (i.e., invention), new techniques using existing technologies, and improved preoperative and postoperative patient-care practices. For our purposes, we focus on research with the potential and intent to immediately alter perioperative and/or intraoperative patient care.

Although the field of surgery is deeply rooted in tradition, surgeons refine their own “best practices” while navigating the complexity of human anatomy. Therefore, the heart of surgical innovation lies within the surgeon. Although surgeons hold intimate understanding of human anatomy, the nuances of their operating rooms and the limitations of current surgical treatment, surgical innovation is often not a focus for academic and community surgeons.^3^ While surgical departments value research, individual surgeons feel that it is not their role to innovate.^4^ With many competing academic duties, there is little incentive for surgeons to engage in the formal innovation process.

In the business model, idea generation is thought to be the biggest hurdle for product innovation.^2^ Surgeons frequently generate ideas for improvement as they encounter frustrations during surgery, but these ideas rarely leave the operating room. When idea generation seems to be the only phase that surgeons have mastered, why is innovation not incorporated into a surgeon’s career? A 2017 editorial^3^ in the *Canadian Journal of Surgery* suggested that, like the United States, a lack of funding may be a barrier. Funding for surgical departments from the National Institutes of Health (NIH) in the United States dropped from 3.0% to 2.3% of all public research funding in less than one decade.^5^ The objective of our study was to evaluate the trend in funding for surgical innovation in Canada over the past decade. Specifically, we calculated funding acquired by surgical departments from the Canadian Institutes of Health Research (CIHR) between 2008 and 2019, as well as the proportion of funding that was dedicated towards surgical innovation.

## Methods

To understand recent trends in public research funding that is allocated to surgical innovation in Canada, we searched the CIHR Funding Decisions Database for successful grants between 2008 and 2019. The database includes all successful applications, regardless of whether the award was accepted by the applicant. To capture grants of interest, all studies under the “Department of Surgery” were screened. A comprehensive search of each of the subspecialty surgery departments listed on the CIHR search engine was performed to capture grants not filed under “Department of Surgery.” The listed surgical subspecialties included “Department of Otolaryngology,” “Department of Vascular Surgery,” “Department of Neurosurgery,” “Department of Plastic Surgery,” “Department of Urology,” and “Department of Orthopedics.” The “Department of Obstetrics and Gynecology,” “Department of Gynecology and Medicine,” and “Department of Medicine and Obstetrics and Gynecology” were also searched. The titles and abstracts were screened for surgical innovation by two independent reviewers (RW and KK) using the inclusion and exclusion criteria. Any discrepancies noted between the two reviewers were resolved through consensus discussion. A third reviewer (MSS) was involved if no resolution was reached.

The inclusion criteria were those with a focus on novel perioperative optimization strategies (i.e., changes to patient care in the pre- and postoperative periods, including preoperative decision-making tools and postoperative pain management or anticoagulation regimens), and/or intraoperative techniques or equipment. Exclusion criteria included: 1) conservative or medical management of a disease, 2) understanding molecular mechanisms of surgical disease states, 3) preclinical phase of intervention, 4) non-surgical disease state or obstetrical presentations 5) health systems/quality improvement, 6) summer studentships, and 7) no abstract available/included.

We excluded basic science research that seeks to understand surgical diseases, as a translational gap often exists between this understanding and surgical innovation.^2^ However, we included basic science research with the intent to directly improve surgical care. For example, we included a study of biomechanical factors of spinal cord injury in which the findings were related to optimal timing of surgical decompression.

The total amount of surgical innovation funding in each calendar year was calculated and adjusted, based on inflation rates derived from the Bank of Canada website, to reflect the 2019 Canadian dollar value. The total yearly research funding (not limited to surgical innovation research) allocated to surgical specialties were also derived for comparison.

## Results

### Search Process

**Figure 1.**
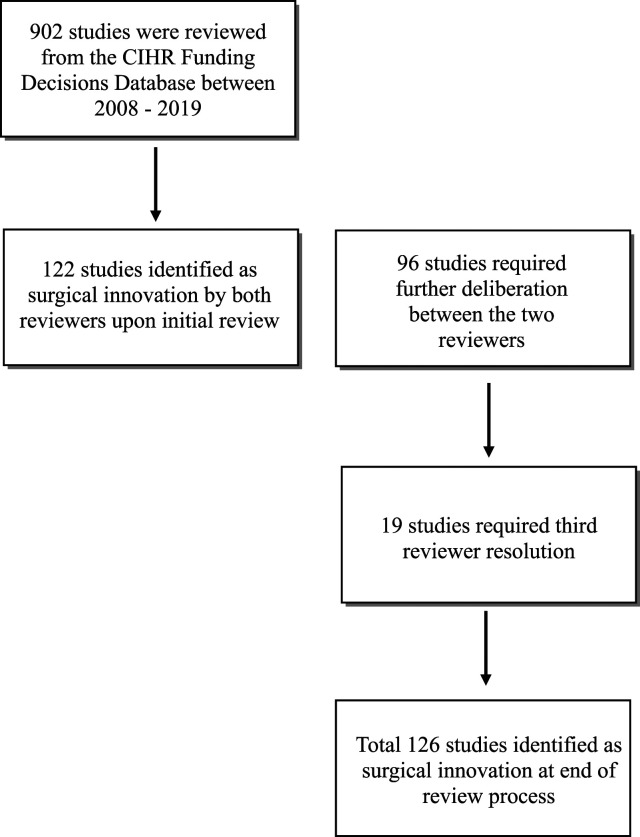
Flowchart demonstrating the CIHR Funding Decisions Database review process. Out of the 902 studies screened, 126 studies were identified to have a focus on surgical innovation.

A total of 902 titles/abstracts were reviewed from the CIHR Funding Decisions Database from all surgical departments (Figure 1). Among these, 122 studies were immediately identified as having a surgical innovation component by both reviewers, but 96 studies required additional deliberation. Nineteen studies required a third reviewer for resolution. At the end of the review process, 126 studies met criteria for research focused on surgical innovation in Canada. These studies originated from 26 different university-affiliated organizations.

We organized the 126 studies into 1 of 4 categories including: a) promotion of preoperative shared decision-making (e.g., “development of decision tool in post-mastectomy breast reconstruction recovery and risk stratification of endometrial cancer to inform surgical management”), b) optimization of surgical technique or practice (e.g., “advanced real-time 3D imaging of fracture surgery and regeneration of transplantable human lungs from native matrix scaffold”), c) optimization of postoperative outcomes (e.g., “advanced allograft monitoring in human lung transplant and integrating mobile app to reduce readmission following colorectal surgery”), and d) reduction of perioperative mortality and morbidity (e.g., “implantable device for early detection of orthopedic infection and prevention of delirium in postoperative cardiac patients through novel markers).”

### Funding in Surgical Innovation

**Figure 2.**
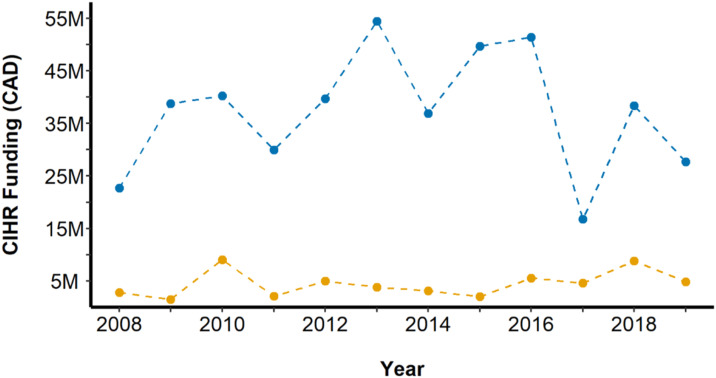
Amount of CIHR funding allocated to surgical departments (blue) and to surgical innovation research (yellow) in Canada between 2008 and 2019, adjusted for 2019 inflation. Total funding allocated to surgical departments ranged between 16.8 million and 54.4 million per year. Total funding allocated to surgical innovation research ranged between 1.5 million and 9.1 million per year.

Between 2008 and 2019, there was an upward trend in CIHR funding allocated to surgical innovation, despite no distinct trend in funding allocated to surgical departments overall (Figure 2).

Total funding allocated to surgical departments ranged between 16.8 million and 54.4 million per calendar year. The amount allocated to surgical innovation research ranged between 1.5 million and 9.1 million per calendar year. During the nadir of total funding allocated to surgical departments in 2017, where grants totaled 16.8 million, surgical innovation funding remained at 4.6 million.

**Figure 3.**
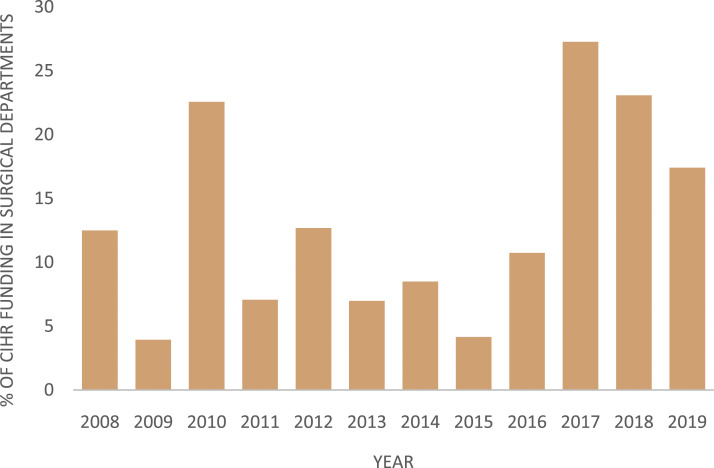
Percentage of CIHR grants allocated to surgical departments focusing on surgical innovation ranged from 3.9% to 27.3%. Between 2008 and 2019, the total funding allocated to surgical departments ranged between 16.8 million and 54.4 million per year.

**Figure 4.**
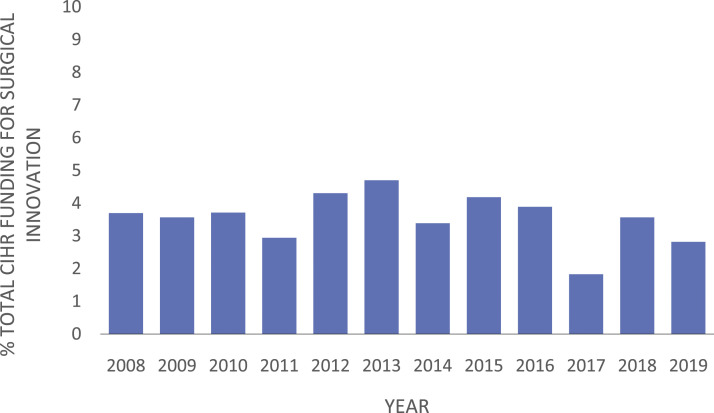
Percentage of total CIHR funding from 2008 to 2019 allocated to the surgical departments in Canada, which ranged from 1.82% to 4.7%. Total CIHR funding ranged between 519 million and 1.2 billion.

The percentage of total funding granted to surgical innovation research ranged from 3.9% (1.5 million of 38.7 million) to 27.3% (4.6 million of 16.8 million) (Figure 3). The most recent 3 years had a higher percentage of the total department funding allocated toward surgical innovation. The percentage of total CIHR funding awarded to surgical departments ranged between 1.8% (16.8 million of 920.4 million) to 4.7% (54.4 million of 1.1585 billion) (Figure 4).

## Discussion

The objective of our study was to determine whether the perceived lack of surgical innovation coming out of Canada can be explained by decreasing public funding support.^3^ Over the last decade, innovation in perioperative and intraoperative patient care has received increasing attention, as demonstrated by the national funding allocated to surgical departments.

Depending on the year, between 3.9% and 27.3% of the total public funding for surgical departments was awarded to surgical innovation in perioperative and intraoperative patient care. However, the overall percentage of CIHR funding awarded to surgical departments, as compared to non-surgical departments, remains low at less than 5%. This proportion is comparable to data from the United States NIH grants, where non-surgical departments, including internal medicine, pediatrics, psychiatry, pathology, and microbiology, received more funding than surgical departments.^6^ Based on these data, it is difficult to conclude whether the small proportion of funding for surgical innovation is due to the limited funding available to surgical departments or due to surgeons not applying or qualifying for CIHR grants.

It is important to acknowledge that surgeons face numerous barriers to obtaining national research grants. For example, unless a surgeon holds a formal academic relationship and position with their home university, there is often little protected research time for innovation. Surgeons at academic and community sites have many responsibilities, including clinical duties, teaching commitments, and administrative obligations. Many surgeons lack the entrepreneurship background or training to know where to begin. Other challenges may come from higher organizational levels of monitoring and regulation posing insurmountable barriers.^7^ In short, many surgical departments are not part of an ecosystem of innovation. A local innovation ecosystem is defined as having local access, funding, policies, and a culture that fosters collaboration, consultation, and experimentation.^8^

Teaching the fundamentals of innovation and invention processes early in training may translate into interest and aptitude during a surgeon’s career. Formal training in surgical innovation has led to the generation of intellectual property by those with minimal background in entrepreneurship. For instance, teaching models such as the Business Engineering Surgical Technologies (BEST) have trained young professionals across the fields of business, engineering, and medicine to create new patents and industrial collaborations.^9^ These programs can be easily integrated into the medical curriculum if prioritized. Nevertheless, beyond competency, the continued participation in surgical innovation requires additional support through means of funding and infrastructure.^10^ Many public funding systems reward those with a research track record and access to raw data. To inspire innovation, we need more funding that provides opportunity for new investigators taking on high-risk innovative research. For example, the National Institute of Health Director’s New Innovator Award attempts to model this philosophy. Once funding for a proposed innovative idea is secured, the first step in product or process innovation can begin.

While surgeons may be at the forefront of idea generation, successful innovation is ultimately achieved through a collaborative framework with bioengineers. Without an engineer, biomedical engineering theories can become too sophisticated for a surgeon to comprehend in the absence of formal training. Similarly, without a surgeon, the surgical applicability of an engineered product may not be clinically relevant or user friendly. The Surgical Innovations program at the University of California, San Francisco (UCSF) exemplifies the accelerated productivity that results from collaboration between surgeons, bioengineers, and trainees.^11^ Over the past 15 years, this collaboration has further expanded to include early-stage angel investors and later stage venture capital firms. Although not surgical innovation, this successful enterprise has led to inventions such as alternatives to dialysis for people with end-stage kidney disease and magnetic treatment as an alternative to CPAP for obstructive sleep apnea. Similarly, academic training has identified the value of multidisciplinary collaboration. The concept of “Surgineering” has been increasingly implemented in engineering curricula.^12,13^ It emphasizes the cross-pollination of the medical and engineering curricula and allows engineering students to obtain hands-on training from surgeons on basic surgical principles and technologies. Promotion of integrated multidisciplinary teaching early in training ultimately provides a platform to invite future collaboration efforts.

Overall, surgical innovation is a broad term which can be further subcategorized. We focused on understanding how the public sector in Canada supports surgeons in innovation of perioperative and intraoperative patient care practices. We recognize that the innovation ecosystem likely includes funding from the private sector and multidisciplinary sources as well. Future directions can include investigating all funding sources. The data presented here establishes public funding patterns for surgical innovation prior to the COVID-19 pandemic. Future examination of funding for surgical innovation can be compared to the pre-pandemic era.

## Conclusion

Public funding for surgical innovation in Canada has increased over time. However, surgical innovation funding remains a small percentage of the overall funding allocated to medical research. In countries where funding access is not considered a major barrier, we need to prioritize a collaborative multidisciplinary framework with surgeons, engineers, and industry. In addition, we should aim to cultivate aptitude in innovation early in medical training, create protected time and space for surgical faculty to engage in innovation projects, and introduce dedicated funding streams for surgical innovations.
